# Neuroprotective Effects of Quercetin in Pediatric Neurological Diseases

**DOI:** 10.3390/molecules25235597

**Published:** 2020-11-28

**Authors:** Lourdes Alvarez-Arellano, Marcela Salazar-García, Juan Carlos Corona

**Affiliations:** 1 CONACYT-Hospital Infantil de México Federico Gómez, Mexico City 06720, Mexico; lourdes.alvareza@gmail.com; 2 Laboratorio de Investigación en Biología del Desarrollo y Teratogénesis Experimental, Hospital Infantil de México Federico Gómez, Mexico City 06720, Mexico; msalazar.investigacion@gmail.com; 3 Laboratory of Neurosciences, Hospital Infantil de México Federico Gómez, Mexico City 06720, Mexico

**Keywords:** quercetin, oxidative stress, pediatric diseases, neuroprotection

## Abstract

Oxidative stress is a crucial event underlying several pediatric neurological diseases, such as the central nervous system (CNS) tumors, autism spectrum disorder (ASD) and attention-deficit/hyperactivity disorder (ADHD). Neuroprotective therapy with natural compounds used as antioxidants has the potential to delay, ameliorate or prevent several pediatric neurological diseases. The present review provides an overview of the most recent research outcomes following quercetin treatment for CNS tumors, ASD and ADHD as well as describes the potential in vitro and in vivo ameliorative effect on oxidative stress of bioactive natural compounds, which seems like a promising future therapy for these diseases. The neuroprotective effects of quercetin against oxidative stress can also be applied in the management of several neurodegenerative disorders with effects such as anti-cancer, anti-inflammatory, anti-viral, anti-obesity and anti-microbial. Therefore, quercetin appears to be a suitable adjuvant for therapy against pediatric neurological diseases.

## 1. Introduction

Flavonoids belong to a large group of natural polyphenolic phytochemicals which have been shown to produce several effects such as antioxidative and anti-inflammatory [1,2] and several studies have highlighted the potential beneficial role of flavonoids in numerous neurodegenerative diseases [3,4,5]. There are several subclasses of flavonoids which include the flavones (such as luteolin, rutin, chrysin, baicalin and oroxylin A), flavanones (such as naringenin and hesperidin), isoflavones (such as daidzein and genistein), proanthocyanidins (such as procyanidins), flavanols (such as catechin and epicatechin) and flavonols (such as kaempferol, myricetin and quercetin).

Quercetin is a flavonoid compound present in a wide variety of vegetables and fruit, such as onion, asparagus, red leaf lettuce, cilantro, lovage, dill, capers, apples, and berries. Quercetin represents the highest percentage of total flavonoid intake and is the most important component of flavonol subclass, often the base of other flavonoids [6,7,8]. Thus, quercetin has been demonstrated to exert neuroprotective effects in several neurodegenerative disorders [9,10,11] as well as antioxidant, anti-inflammatory, anti-cancer, anti-obesity, anti-viral and anti-microbial properties, and cardioprotective and hepatoprotective activities [7,8,9,12].

## 2. Antioxidant Effects of Quercetin

The antioxidant effects of quercetin are elicited owing to the presence of several hydroxyl groups and the basic flavonol skeleton. Quercetin is a potent scavenger of reactive oxygen species (ROS), including superoxide, peroxynitrite and hydrogen peroxide (H_2_O_2_), which is also a good lipid peroxidation inhibitor [8,13,14]. One of the antioxidant effects of quercetin depends on the level of glutathione (GSH). Thus, high GSH level promotes the formation of the 6-glutathionyl-Qu (GSQ) complex, which enhances the antioxidant effect, while a low level of GSH increases the extent of cellular damage [7,15].

The effects of quercetin against oxidative stress have been widely demonstrated in diverse conditions both in vitro and in vivo. Accordingly, it was shown that quercetin reduces the H_2_O_2_-mediated oxidative stress in yeast mutant cells [16]. Oxidative stress plays a role in the pathophysiology of mental diseases, such as depression or anxiety. Repeated predator stress exposure to mice produced freezing, anxiety-like and depressive-like behaviors. Quercetin showed a protective effect against depression and could alleviate the fear of traumatic events in these mice [17]. Treatment with quercetin showed a protective effect against the oxidative stress produced by cadmium exposure in rats via decrement of the malondialdehyde (MDA) content and an increment in the levels of antioxidant enzymes, superoxide dismutase (SOD), glutathione peroxidase (GPx) and catalase (CAT) [18]. Quercetin protected against doxorubicin-induced cardiomyopathy in rats by increasing the levels of antioxidant defense molecules such as the nuclear factor erythroid 2-related factor 2 (Nrf2), which is a regulator of cellular defense against oxidative stress as well as via the restoration of histological and biochemical defects [19]. Lipopolysaccharide-induced intestinal oxidative stress exerted in broiler chickens and quercetin could significantly inhibit oxidative stress and up-regulate the SOD and GPx levels. Moreover, quercetin relieved mitochondria damage and up-regulated mitochondrial DNA copy number-related gene expression. Furthermore, quercetin promoted Nrf2 activation and increased the gene expression level of heme oxygenase-1 (HO-1), NAD(P)H quinone dehydrogenase 1 (NQO1), and manganese (Mn) SOD2 [20]. Furthermore, quercetin protected against oxidative stress induced by bisphenol-A in rat cardiac mitochondria, considering the improved mitochondrial membrane potential (∆Ψm), GSH level and CAT activity [21]. In addition, quercetin reduced the generation of ROS and nitric oxide (NO) induced by cigarette smoke exposure both in vitro and in vivo, as well as reduced the levels of oxidative stress, leukocyte level and histological pattern changes of the pulmonary parenchyma [22]. Finally, it was demonstrated that quercetin could ameliorate diabetic encephalopathy in mice as a result of reduction in the learning and memory dysfunction, reduced fasting blood glucose and increased insulin sensitivity; in addition, quercetin inhibited oxidative stress and ameliorated neurodegeneration. Moreover, quercetin activated SIRT1, which is an enzyme that deacetylates proteins, which contribute to cellular regulation and inhibited the expression of the endoplasmic reticulum (ER) stress-related proteins [23].

## 3. Central Nervous System (CNS) Tumors

CNS tumors are a heterogeneous group of neoplasms representing the primary cause of death among children and adolescents. Among all brain tumors, glioblastoma, medulloblastoma, and ependymoma are the most common ones in pediatric populations [24,25,26]. Gliomas are tumors that originate from the glial cells (the supporting cells of CNS) and exists in diverse types based on the involved cell type, for example, astrocytoma, glioblastoma, ependymoma, oligodendroglioma and oligoastrocytoma [24]. Despite the conventional therapy including surgical resection followed by chemotherapy (mainly with temozolomide) and radiotherapy, the effectiveness of this treatment approach is extremely limited and the prognosis is poor with a median survival of 1 year after the therapy. In addition, the most common side-effects of this therapy include cognitive and endocrine dysfunctions, as well as secondary malignancies [25]. Therefore, new natural therapeutic strategies are warranted for use either alone or in combination with other pharmacological agents for the treatment of CNS tumors. Until date, multiple studies have demonstrated the antitumor effect of quercetin on different types of cancer, including breast, esophageal, colorectal, stomach, prostate, lung, ovarian, melanoma and leukemia [27,28,29,30,31]. In addition, quercetin has been reported to induce angiogenesis, the inhibition of proliferation, metastasis, chemoresistance as well as apoptosis both in vitro and in vivo [32].

### 3.1. Protective Effects of Quercetin in CNS Tumors

The growth of brain tumors induces an increase in the level of ROS and damage of non-malignant cells, which leads to apoptotic cell death and the production of necrotic areas. This niche creates a favorable microenvironment for the existence of stem cells responsible for the resistance and recurrence of the tumor. The necrotic areas also favor chemo- and radio-resistance, which correlates with a poor prognosis for survival [33,34]. The potential benefits of the antioxidant activities of quercetin in CNS tumors remains controversial and not sufficiently addressed. In a recent study, quercetin-loaded nanoparticles were applied to rat glioma C6 cells that resulted in decreased cell proliferation and MDA levels, which in turn decreased the oxidative stress [35]. Moreover, quercetin pre-treatment in stressed U-87MG cells with H_2_O_2_ or glucose oxidase enhanced the levels/activities of SOD1, GSH and CAT; however, quercetin decreased the SOD2 level but increased the SOD2 activity as well as decreased the cyclooxygenase-2 (COX-2) expression and increased the apurinic/apyrimidinic endonuclease 1 (APE1) expression in the stressed cells [36]. However, recently, it was found that quercetin and chloroquine can activate stress to both the mitochondria and ER-promoting glioma cell death [37]. In another study, high concentrations of quercetin were recorded in nanoliposomes on C6 glioma cells, which in turn induced a decrease of ∆Ψm, loss of adenosine triphosphate (ATP) and increased ROS production that together resulted in necrotic cell death, although lower concentrations of quercetin can induce apoptosis [38]. In a similar study, PEGylated nanoparticles of quercetin demonstrated dose-dependent cytotoxicity to C6 glioma cells and increase in the ROS levels, which led to the up-regulation of p53 protein and an increase in the cytochrome C and caspase-3 protein levels [39]. Conversely, increases in the caspase-3, 8 and 9 enzyme activities were identified in H_2_O_2_-treated C6 glioma cells and that were then blocked by the addition of quercetin, which resulted in the blocking of phosphorylated extracellular signal-regulated protein kinase (ERK) and p53 protein expressions induced by H_2_O_2_. Also, in C6 glioma cells quercetin acted antagonistically on arsenic trioxide-induced growth inhibition and also increased GSH levels [40]. Thus, quercetin exhibited an inhibitory effect on both ROS-independent and -dependent cell death, with the involvement of the induction of HO-1 protein expression [41]. These findings together suggested that the antioxidant activity-depended antitumor effect of quercetin in glioblastoma is largely depends on the concentration of the flavonoid.

On the other hand, an inflammation in the tumor microenvironment is critical for the initiation and progression of glioblastoma [42]. Thus, a high degree of infiltration and activation of immune cells as well as the secretion of inflammatory cytokines in the glioblastoma microenvironment has been demonstrated [43]. Therefore, quercetin is also an important mediator of inflammation and oxidative stress in the CNS [7]. The administration of quercetin reduced the production of neuroinflammation Mn-induced via the inhibition of the expression of inflammatory markers such as COX-2, tumor necrosis factor-α (TNF-α), interleukin-6 (IL-6), IL-1β, and inducible NO synthase (iNOS) as well as decreased ROS and protein carbonyl levels and increased SOD1 activity induced in Mn-treated rats [44]. In T98G and U87 glioblastoma cells, quercetin acted as a potent inhibitor of the IL-6-induced STAT3 signaling pathway [45].

### 3.2. Effects of Quercetin on Tumor Cell Death

One of the main effects of quercetin on glioblastoma is the induction of diverse types of cell death. Apoptosis is a type-I programmed cell death that can be induced via extrinsic and intrinsic pathways. The extrinsic or mitochondrial pathway is mediated by B-cell lymphoma 2 (Bcl-2) family proteins, while the intrinsic pathway is triggered through the activation of death receptors involving caspase-8 activation. Both the pathways converge in the execution phase that involves the activation of caspase-3 [46]. Quercetin induces apoptosis in human U373MG, T98G and rat C6 cell lines as a result of induction of cytochrome C release, a decrease in the level of Bcl-2 and ∆Ψm and an increase in the level of Bcl-2-associated X protein (Bax) along with proteolytic activation of caspase-3 and 7 [47,48]. In addition, quercetin has been demonstrated to potentiate the effect of drugs used in the treatment of glioblastoma such as temozolomide. Thus, combined treatment with quercetin and temozolomide induced apoptosis in T98G cells by triggering cytochrome C release and a decrease of ∆Ψm [49]. Moreover, quercetin has been reported to sensitize glioblastoma cells to temozolomide via the inhibition of the expression of molecular chaperone heat shock protein 27 (Hsp27), which regulates apoptosis [50]. In accordance, the simultaneous administration of quercetin and imperatorin induced apoptosis more effectively than a single drug in glioblastoma T98G cells. As a result, an increase in the activities of caspase-3 and caspase-9 and a decrease in the expressions of Hsp27 and Hsp72 were observed [51]. Similarly, the simultaneous administration of quercetin and sorafenib induced apoptosis in human anaplastic astrocytoma and glioblastoma multiforme cell lines [52].

Autophagy (also known as type-II programmed cell death) in carcinogenesis plays a controversial role because at the initial stage of carcinogenesis, autophagy can prevent the formation of tumor and its progression. However, at the advanced stage, autophagy acts as a defense mechanism to promote the survival of tumor cells, which results in the development of resistance to chemotherapy. Furthermore, autophagy can prevent tumor cells from dying during nutritional deprivation by inhibiting apoptosis [53,54]. Past studies have shown quercetin treatment inhibits t-AUCB-induced apoptosis by increasing the expression of Atg7 (activation of autophagy) and inhibiting COX-2 and Hsp27 in U251 and U87 cell lines and in a xenograft mouse model [55,56]. Moreover, the induction of autophagy in U373MG glioblastoma cells by quercetin was confirmed through the conversion of LC3-II and acridine orange staining [47]. Interestingly, quercetin inhibited cell viability and induced autophagy of U87 and U251 glioma cells in a dose-dependent manner. Thus, the suppression of autophagy at a later stage enhanced the anti-glioma efficiency of quercetin. In contrast, the inhibition of early stage autophagy attenuated the quercetin-induced cytotoxicity [57]. The outcomes of the protective effects of quercetin in CNS tumors are summarized in Table 1.

On the other hand, in a rat glioma model, the daily intraperitoneal administration of quercetin increased the tumor volume in a time-dependent manner. Moreover, a small reduction in lymphocytic infiltration, which is an indicator of good prognosis, and a small reduction of T lymphocyte proliferation were observed [58]. A recent study showed that quercetin decreased the intracellular pH in a mouse model of glioblastoma multiforme [59]. These changes in the intracellular pH are critical considering that alkaline pH favors cell proliferation, invasion, chemoresistance and apoptosis evasion [60,61,62,63]. Interestingly, quercetin used in monotherapy at an extremely low concentrations did not affect cell proliferation, while its combination with irradiation showed decreased medulloblastoma cell line growth and increased survival in orthotopically xenografted mice. Moreover, quercetin did not affect the proliferation of normal human fibroblasts or neural precursor cells [64]. Another important finding is that flavonoids (such as quercetin, kaempferol and myricetin), at concentrations achievable through the consumption of a typical diet, inhibited medulloblastoma cell migration via inhibition of the tyrosine kinase receptor Met or Met-induced activation of Akt, as well as by avoiding the formation of actin-rich membrane protrusions [65]. Therefore, further studies are warranted along with, subsequently, a greater number of clinical trials to demonstrate the effect of quercetin in CNS tumors as an adjuvant therapy in the currently practiced treatment regime.

**Table 1:** Summary of protective effects of quercetin in CNS tumors, ASD and ADHD.

**Type of Study in CNS Tumor**	**Effects**	**References**
Human glioblastoma and rat glioma cell lines	Reduced cell proliferation and increased antioxidant system	[35,36,40]
Rat glioma and human glioblastoma cell lines	Induced cell death due to increased oxidative stress and activation of caspases	[37,38,39,47,48]
Glioblastoma cell lines	Anti-inflammatory activity by inhibition of the STAT signaling pathway	[45]
Glioblastoma and astrocytoma cell lines	In combination with other compounds induced apoptosis	[49,50,51,52]
Mouse model glioblastoma and cell line	Induced autophagy by LC3-I processing and dose-dependency	[47]
Rat glioma model	Increased tumor volume and reduced T lymphocyte infiltration and proliferation	[58]
Medulloblastoma cell lines and mouse model	Decreased cell migration and growth tumor and increased survival	[64,65]
**Type of Study in ASD**	**Effects**	**References**
Children	Safe, well-tolerated and with a positive impact through reduction of brain and gut inflammations	[66]
Children	In an open-label pilot study, it effectively reduced symptoms without any adverse effects	[67]
Developmental hypothyroidism rat model	Recovered expression of NQO1 and Txn1, restored NeuN-positive granule cells, parvalbumin and somatostatin-positive interneurons and recovered the expressions of Otx2 and Gria3	[68]
Prenatal model in rats induced by valproic acid	Prevented behavioral changes, alterations in total thiol content and changes of SOD, CAT and GST in the hippocampus, prevented the alterations of CAT and GPx in the cerebellum, prevented the increase of ROS, nitrite and TBARS levels in the striatum and prevented nitrite and CAT alterations in the cerebral cortex	[69]
**Type of Study in ADHD**	**Effects**	**References**
Children and adolescents	In a randomized controlled trial, it showed clinical benefits and tolerable side-effects	[70]
Children and adolescents	In a randomized double-blind controlled trial of 8 weeks, it did not improve symptoms	[71]
Adolescents	A preliminary study improved some symptoms in patients	[72]
SH-SY5Y cells	Increased ATP levels	[73]
SHR model	Reduced plasma MDA levels, aortic superoxide production and also improved NO-dependent acetylcholine relaxation, inhibited eNOS phosphorylation and reduced the blood pressure	[74]
SHR model	Reduced oxidative stress	[75]
Amphetamine-induced unilateral rotations in rats	Reduced rotations and also attenuated the rotenone-induced loss in striatal dopamine, up-regulated mitochondrial complex-I activity and increased CAT and SOD	[76]
SHR model and H9C2 cells	Prevented cardiac hypertrophy by suppressing AP1 transcription activity and by increasing activation of PPARγ, also the ultrastructural damage of mitochondria and myofibrils were attenuated	[77]
MPH-induced hyperlocomotion in mice	Blocked hyperlocomotion and an increase in lipid peroxidation levels in the striatum and prefrontal cortex regions	[78]

## 4. Autism Spectrum Disorder (ASD)

ASD is a heterogeneous and complex neurodevelopmental condition characterized by significant deficiencies in social interaction, communication and repetitive patterns of behavior [79]. People with ASD spend less time engaged in social interaction when compared with non-ASD individuals. Notably, children with ASD do not attribute sufficient value to potential social interactions and favor other environmental stimuli that seems more valuable to them. The worldwide population prevalence of ASD is approximately 1%, and its onset occurs during childhood before the 3 years of age. Autism affects boys more than girls and it has high comorbidity correlation with other neurological disorders [80,81]. The etiology of ASD involves genetic factors or is associated with Rett Syndrome, Fragile X and Down Syndrome with de novo mutations and also environmental factors (exposure to toxins, neurotoxic metals and smoking) as well as certain types of medications during embryonic development, maternal stress, infections during pregnancy and metabolic- and immune- related nutritional factors [82,83,84,85]. The most common treatment in children and young adults with ASD include the use of antipsychotics and the medications used for ADHD and antidepressants [86,87,88]. However, there is evidence of inconsistent efficacy and significant side-effects for most of these pharmacological interventions [89]. In consequence, there is presently a huge interest in the search for alternative ASD treatments, with natural compounds with demonstrated neuroprotective potential via their antioxidant properties and tolerable side-effects [90].

### Protective Effects of Diverse Compounds That Include Quercetin in ASD

It has been reported that oxidative stress in combination with genetic factors and inflammation could be involved in the pathophysiology of ASD [91,92]. Accordingly, the formulation NeuroProtek that contains the flavone luteolin and the flavonoids quercetin and rutin, was applied in 37 children with ASD. The liposomal formulation was found to be safe and well-tolerated, and it showed a positive impact through reduction of brain and gut inflammations [66]. An open-label pilot study of a formulation containing quercetin, luteolin, and the quercetin glycoside rutin was found to effectively to reduce the ASD symptoms with no major adverse effects recorded [67]. Moreover, the induction of developmental hypothyroidism can be used as a model of ASD and can disrupt hippocampal neurogenesis. A diet containing α-lipoic acid as an antioxidant and α-Glycosyl isoquercitrin (AGIQ), which is a mixture of quercetin glycoside consisting of isoquercitrin and its α-glucosylated derivatives along with >10 additional linear glucose moieties, possesses antioxidant effects. The AGIQ-recovered expression of some antioxidant enzyme genes such as NQO1 and thioredoxin 1 (Txn1) in the developmental hypothyroidism rats also restored of NeuN-positive post-mitotic granule cells, parvalbumin and somatostatin-positive interneurons and both antioxidants recovered expression of GABAergic interneuron-related gene orthodenticle homeobox 2 (Otx2) and also AGIQ-recovered expression of glutamate ionotropic receptor AMPA type subunit 3 (Gria3), thereby reversing the disruptive neurogenesis through compensatory responses [68]. Recently, in an experimental model of autism induced by valproic acid during the gestational period, the prenatal treatment with quercetin prevented the behavioral changes and also the treatment with quercetin prevented alterations in the total thiol content as well as changes in the activities of SOD, CAT and glutathione-S-transferase (GST) enzymes in the hippocampus, which in turn prevented the alterations in the CAT and GPx activity in the cerebellum to thereby prevent an increase in the level of ROS, nitrite and thiobarbituric acid reactive substances (TBARS) levels in the striatum and that in the nitrite and CAT alterations in the cerebral cortex [69]. Thus, compounds that contain quercetin can improve the antioxidant defense mechanism. However, more research is warranted to support the efficacy of quercetin alone or in combination with other flavonoids as a possible treatment option for ASD. The outcomes of the protective effects of quercetin in ASD are summarized in Table 1.

## 5. Attention-Deficit/Hyperactivity Disorder (ADHD)

**Figure 1:**
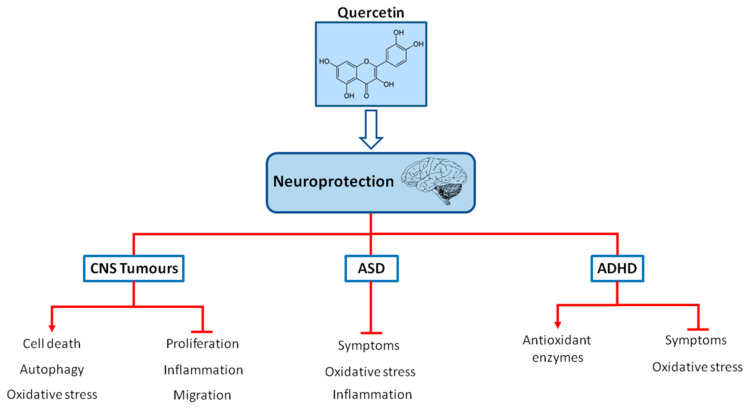
Chemical structure of quercetin and neuroprotection in pediatric neurological diseases (CNS tumors, ASD and ADHD). Quercetin may act as a neuroprotector in pediatric neurological diseases via the regulation of oxidative stress, inflammation, proliferation and improving symptoms and also via increasing antioxidant defenses, autophagy or cell death.

ADHD is the most prevalent neuropsychiatric disorder, with a worldwide prevalence in children of 7.2% [93,94,95]. The characteristic symptoms of ADHD include hyperactivity, lack of attention and impulsivity [96]. In around 50% of children and adolescents diagnosed with ADHD, the symptoms persist throughout the adult life as well [94,97]. The symptoms of ADHD cause problems in personal, scholar, social, or work performance resulting in the consequences of isolation, lower socioeconomic status and increased risk of substance abuse in adolescence, as well as changes of development of comorbidity and antisocial and delinquent behavior [94,97]. Pharmacological treatment with psychostimulants and non-psychostimulants for this condition include the medications aimed at improving the symptoms of ADHD. Methylphenidate (MPH) and amphetamines increase extracellular dopamine and norepinephrine release in the hippocampus, prefrontal cortex, and striatum, which in turn improve neurotransmitter imbalance and symptoms [98,99,100]. The therapy with atomoxetine, which is a selective norepinephrine reuptake inhibitor, increases extracellular dopamine and norepinephrine release in the cerebellum, prefrontal cortex, hypothalamus and hippocampus, resulting in behavioral improvement [100,101,102]. Nevertheless, psychostimulants induce side-effects such as insomnia, appetite loss, headache, abdomen pain, sleep disturbance and anxiety [4,103]. In addition, non-psychostimulants can produce nausea, diarrhea, somnolence, vomiting, appetite loss, fatigue, dizziness, and changes in the cardiovascular events [4,104]. Extensive studies have suggested that the pathophysiology of ADHD is associated with oxidative stress [96,105,106,107]. Therefore, there is an increasing interest in the search for alternative treatments for ADHD, including the application of bioactive natural compounds owing to their antioxidant properties and considering that these alternative treatments options may have minimal side-effects. The neuroprotective mechanisms of quercetin in pediatric neurological diseases are summarized in Figure 1.

### Protective Effects of Diverse Compounds That Include Quercetin in ADHD

As indicated earlier, accumulating evidence indicate that oxidative stress is involved in the pathophysiology of ADHD [96,105,106,107] and also that the administration of MPH can induce oxidative stress in neurons and thereby neurodegeneration in the cerebral cortex and the hippocampus of animals [108]. Passionflower, which is commonly known as Passiflora incarnata, contains quercetin and other ingredients. In a randomized controlled trial with passionflower in children and adolescents with ADHD, significant clinical benefit and a tolerable side-effect profile was achieved as result of the advantages of passionflower as compared with MPH [70]. St. John’s wort (Hypericum perforatum extract) that contains quercetin among other flavonoids, was used in a randomized double-blinded controlled trial of 8 weeks-duration, but it showed no improvement in the ADHD symptoms [71]. Conversely, a preliminary study demonstrated that treatment with St. John’s wort improved some symptoms in ADHD patients [72]. In vitro, the treatment with St. John’s wort could significantly increase the ATP levels in SH-SY5Y cells [73]. The outcomes of the protective effects of quercetin in ADHD are summarized in Table 1.

The spontaneously hypertensive rat (SHR) is presently used as a validated animal model of ADHD [109]. It was demonstrated that the treatment of SHR with quercetin could reduce its plasma MDA levels and aortic superoxide production as well as improve NO-dependent acetylcholine relaxation, which inhibited endothelial NO synthase (eNOS) phosphorylation and reduced the blood pressure [74]. It was also observed that an increase in oxidative stress in SHR, could be reversed by the treatment with quercetin [75]. Moreover, treatment with quercetin in rats showed significant reduction in the amphetamine-induced unilateral rotations, attenuation of rotenone-induced loss in striatal dopamine, up-regulation of the mitochondrial complex-I activity and increase in the CAT and SOD levels [76]. Quercetin prevented cardiac hypertrophy via suppression of the activator protein 1 (AP1) transcription activity and promotion of the activation of peroxisome proliferator-activated receptor γ (PPARγ). Moreover, the ultrastructural damage of mitochondria and myofibrils in both the SHR and H9C2 cells were found to be attenuated [77]. It was previously demonstrated that PPARγ activation has neuroprotective and antioxidant effects [110]. Finally, chronic treatment with quercetin blocked MPH-induced hyperlocomotion and also blocked the increase in lipid peroxidation levels in the striatum and prefrontal cortex regions [78]. Thus, compounds that contain quercetin could improve the neuroprotection through the activation of antioxidant pathways and by its powerful scavenging properties. Nevertheless, further studies are warranted to verify the efficacy, effects, and dosages of quercetin either alone or in combination with other flavonoids as a possible treatment alternative agent against oxidative stress in ADHD.

## 6. Protective Effects of Other Flavonoids in Pediatric Neurological Diseases

There are increasing data with flavonoids to verify their efficacy and potential for CNS tumors treatment. Furthermore, it was demonstrated that flavonoids combined with anticancer drugs led to the enhanced anticancer effect. Thus, flavanols such as epigallocatechin gallate which is a constituent of green tea, alone or in combination with temozolomide inhibited neurosphere formation and cell migration of glioma stem-like cells and the treatment whit epigallocatechin gallate, also affected both migration and adhesion of medulloblastoma cells [111,112]. Besides, the flavone chrysin and the combination with cisplatin, induced apoptosis, cell cycle arrest and ∆Ψm loss in human glioma cells [113]. The flavonoid luteolin significantly inhibited glioma cell proliferation, induced apoptosis via MAPK and caspase activation and promote autophagy [114,115]. Moreover, luteolin induced ER stress and mitochondrial dysfunction leading to cell death in glioblastoma cell lines and in an animal model [116]. The combination of the flavonoids luteolin and silibinin effectively blocked angiogenesis and survival pathways leading to induction of apoptosis [117]. Also, the same combination of flavonoids, induced inhibition of growth of glioblastoma cells by the induction of apoptosis and the inhibition of invasion and migration [118].

Alternative approaches with flavonoids are on continuous research to confirm their efficacy and to understand its potential in ASD treatment. The green tea extract (*Camellia sinensis*) is an important source of flavonols, such as catechins, epicatechin, epigallocatechin and epicatechin-3-gallate and flavonol derivatives such as kaempferol, quercetin and myricetin [119]. Thus, the green tea extract treatment demonstrated amelioration of behavioral and oxidative stress aberrations in an animal model of valproate-induced autism [120]. The treatment of co-ultramicronized palmitoylethanolamide and the flavonoid luteolin in a murine model of autism was efficient in ameliorating social and non-social symptoms via modulation of TNFα and IL-1β immunoreactivity, reduction of GFAP, NF-κB and increased neurogenesis and neuroplasticity in the hippocampus [121]. Moreover, consumption of epigallocatechin-3-gallate, the major compound of catechin in green tea can reverse the behavioural alterations in the sodium valproate-induced autism rat model possibly due to antioxidant effects [122]. Naringenin is a flavanone abundantly found in oranges, grapefruit, and tomato skin. The administration orally for 29 days of naringenin, significantly restored behavioral and biochemical deficits in ASD phenotype in rats induced by propanoic acid [123]. Ginkgo biloba leaves, contains flavonoids (quercetin, kaempferol, and isorhamnetin), terpenoids, and ginkgolic acid. In an observational study of three patients treated with Ginkgo biloba extract, improved aberrant behavior and symptoms of autism [124]. In a double-blind placebo-controlled trial, ginkgo biloba extract was used in patients with autism and the results demonstrated ginkgo biloba no shown significant improvement in the treated group; however, ginkgo biloba was relatively safe and well-tolerated [125].

A growing interest in alternative treatments for ADHD include the research with diverse flavonoids due to their antioxidant properties and because they have minimal side-effects [4]. Baicalin, a major flavonoid isolated from *Scutellaria baicalensis Georgi*, has antioxidative properties. Thus, baicalin regulated the core symptoms of ADHD and also, improved LDH activity and the synaptosomal ATPase via regulating the AC/cAMP/PKA signaling pathway in the SHR [126,127]. Pycnogenol has antioxidants effects and is extracted from French maritime pine bark (*Pinus pinaster*), the main ingredients are procyanidins which are a class of flavonoids and phenolic acids. In a randomized, double-blind, placebo-controlled trials, pycnogenol normalized total antioxidant status, catecholamine concentration, reduced oxidative stress, improved hyperactivity and attention in children with ADHD [128,129]. Moreover, the treatment with pycnogenol improved attention, visual-motor coordination, concentration and also reduced significantly the hyperactivity in children with ADHD [130]. Oroxylin A is a flavonoid found in plants *Scutellaria baicalensis*, *Scutellaria lateriflora* and the *Oroxylum indicum* tree. Oroxylin A has activity as a dopamine reuptake inhibitor and is an antagonist of the GABA-A receptor and also has antioxidant effects. Treatment with oroxylin A and a derivate of oroxylin A, improved ADHD-like behaviors in the SHR [131,132].

## 7. Conclusions

The increasing evidence about the association of pediatric neurological diseases and oxidative stress may play a role in the pathophysiology of CNS tumors, ASD and ADHD. Accordingly, the use of flavonoids such as quercetin as discussed so far has emerged as a promising alternative for therapy against oxidative stress in pediatric neurological diseases. Quercetin can improve CNS tumors, ASD, and ADHD progression conditions due to its antioxidant properties. However, the results of using quercetin need further research that include pretrial dose-finding trials and characterization of individual unique oxidative processes occurring in different pediatric neurological diseases. In addition, quercetin therapy may be required to be administered early in chronic insidious pediatric neurological diseases so as to achieve an appreciable clinical benefit in a timely manner. Intervention in at-risk individuals and pre-disease screening may also be considered in the future. In summary, bioactive natural compounds such as quercetin seem suitable for adjuvant therapy against pediatric neurological diseases.
